# Health-care seeking for childhood diseases by parental age in Western and Central Africa between 1995 and 2017: A descriptive analysis using DHS and MICS from 23 low- and middle-income countries

**DOI:** 10.7189/jogh.11.13010

**Published:** 2021-08-10

**Authors:** Lisa Bogler, Ann-Charline Weber, John Ntambi, Aline Simen-Kapeu, Noel Marie Zagre, Rene Ehounou Ekpini, Sebastian Vollmer

**Affiliations:** 1 Department of Economics and Centre for Modern Indian Studies, University of Göttingen, Göttingen, Germany; 2 United Nations Children’s Fund (UNICEF), West and Central Africa Regional Office, Dakar, Senegal; 3 UNICEF Area Representative for Gabon and São Tomé and Príncipe and to the ECCAS, Libreville, Gabon

## Abstract

**Background:**

Globally, health care seeking for childhood diseases seems to be on the rise. However, progress is slow and still, many cases of infectious diseases in children remain untreated, leading to preventable child mortality. A better understanding of care seeking behaviour may help to further increase the probability that a sick child is taken to a health facility for care.

**Methods:**

We investigated whether mother’s and father’s age at birth of the child is associated with health care seeking behaviour for childhood diseases and how this association changed over time. For this observational study, we used repeated cross-sectional data, namely all available Demographic and Health Surveys as well as Multi-Indicator Cluster Surveys from Western and Central Africa, 1995 to 2017. We analysed care seeking behaviour for diarrhoea, acute respiratory infections (ARI), and treatment of diarrhoea with oral rehydration solution (ORS). We estimated ordinary least squares regressions, controlling for socioeconomic characteristics of the household and adding survey year- and country-fixed effects. Estimated associations are presented for the entire region and for each country separately to highlight heterogeneity.

**Results:**

Overall, the likelihood that care is sought for a child suffering from diarrhoea or ARI is low in Western and Central Africa. Probability of care seeking for diarrhoea ranges between 49% for mothers above 40 years and 53% for mothers between 25 and 29 years. For ARI, the rates are 60% and 62%, respectively. Treatment of diarrhoea with ORS is even lower, ranging between 23% and 26%. The probability that parents seek health care for their child does not seem to be associated with parents’ age at birth. Mother’s level of education and household’s wealth status seem to be more important factors. There is evidence of the relationship between parents’ age and care seeking changing over time, suggesting a stronger association in the past.

**Conclusions:**

Parents’ age at child birth does not seem to have a relevant association with care seeking for common childhood diseases. Identifying relevant factors may help in improving health care seeking behaviour of parents in low- and middle-income countries leading to reductions in child morbidity and mortality.

In 2018, infectious diseases remained a leading cause of under-five mortality. Pneumonia, diarrhoea and malaria accounted for almost a third of global under-five deaths, totalling about 1.49 million deaths among children aged 1-59 months in 2018 [1]. Despite progress over the past decades, the prevalence of these three childhood diseases remain high in many countries [2-4]. This seems surprising given that most of these cases are preventable or treatable at low cost, for example by using insecticide-treated bed nets to prevent malaria or by taking oral rehydration solution (ORS) to treat acute diarrhoea. Globally, health care seeking for childhood diseases seems to be on the rise, meaning that more caretakers bring their ill children to health facilities [2,5,6]. Nationally representative household surveys conducted between 2015 and 2017 in sub-Saharan Africa suggest that 36 percent of children with fever were taken to public health facilities [2]. The proportion of children taken to health care providers for diarrhoea increased in 34 of 37 low- and middle-income countries with data available and the rate of ORS use increased in 36 of 47 countries [5]. However, progress is slow [3]. Yearly increments for the proportion of children taken to health care providers for diarrhoea have been estimated at 2.5 percent [5]. A better understanding of care seeking behaviour is required to reduce preventable child deaths. According to the World Malaria Report 2018, the percentage of children with fever who are brought to health facilities differs greatly between children from households with different socioeconomic characteristics [2]. The gap in care seeking for fever is more than 10 percentage points between the wealthiest and poorest households. Similar findings are reported for pneumonia care seeking [3]. Many studies find evidence of associations between socioeconomic characteristics and health care seeking behaviour, although the strength of the association varies considerably between countries.

Malqvist et al. found lower probabilities of seeking care for children with fever, cough or diarrhoea among less educated mothers and poorer households in Nepal, with parents from the poorest quintile only half as likely to seek care for children than in the richest quintile [7]. In Guatemala, education had little effect on choices for treating acute respiratory infections and diarrhoea in 1995 [8], while it improved management of child diarrhoea in a study some years later [9]. In Mozambique, the probability of seeking care for children with fever was found to be higher among more educated mothers (odds ratio of 4.02 for secondary vs no education) and among richer households (odds ratio of 2.27 in richest vs poorest quintile) [10]. Children from wealthier households were also more likely to seek medical treatment for fever, cough or diarrhoea in Ethiopia (odds ratios of 1.84, 2.46, and 2.46, respectively for richest vs poorest quintile) [11]. Aremu et al. analysed the association between socioeconomic characteristics and treatment choices for childhood diarrhoea in several sub-Saharan African countries [12]. They found that higher parental education was associated with a higher likelihood of treatment compared to no treatment (55.9 percent of children of caregivers with no education receiving no treatment vs 38.6 percent of caregivers with secondary or higher education).

One concern with these studies is the limited comparability of results due to differences in the definition of health care seeking. The main objective of this study is to address this gap with an analysis of the association between parental characteristics as well as household wealth status and health care seeking behaviour that is comparable across countries. We specifically focus on mother’s and father’s age at birth of the child, a factor not highlighted in other studies. The focus on age is partly motivated by evidence that adolescent maternity was still highly prevalent in the 2010s in Western and Central Africa [13]. We assess the hypothesis that very young and very old parents are less likely to seek care for their child compared to parents in a medium age range. In addition, we investigate whether this association changed over time. We used Demographic and Health Surveys (DHS) as well as Multiple Indicator Cluster Surveys (MICS) for all Western and Central African countries from 1995 to 2017. We included care seeking behaviour for diarrhoea, acute respiratory infections (ARI), and treatment of diarrhoea with ORS and investigated country heterogeneities.

## METHODS

### Data

For this observational study, we used cross-sectional, nationally representative survey data from Western and Central Africa. We combined two data sources, the Demographic and Health Surveys (DHS) and Multiple Indicator Cluster Surveys (MICS). The DHS is administered by ICF International. In seven rounds since 1984, the DHS Program has collected nationally representative data in low- and middle-income countries. MICS is a data collection initiative by UNICEF and has collected data on women and children in low- and middle-income countries since 1995. In both survey programmes, women in reproductive age, typically between 15 and 49, were asked for information on all children ever born to them. The surveys apply a multistage stratified sampling design for the within-country selection of households. For each country, regions were defined, within which the population was stratified into urban and rural. For each stratified area, enumeration areas were randomly drawn and denoted as primary sampling units (PSUs). Selection of PSUs was based on a probability proportional to size, in this case the number of households. Within each PSU, all households were listed from the most recent population census. Applying an equal probability systematic sampling, a fixed number of households in each PSU was selected for an interview. Weights for the calculation of nationally representative statistics are provided with the survey data. For the analysis across countries, we re-scaled weights using each country’s female population aged 15 to 49 years in 2017 or the last year of the considered time period [14]. This ensures that small countries do not excessively influence the estimation of overall disease prevalence.

**Figure 1:**
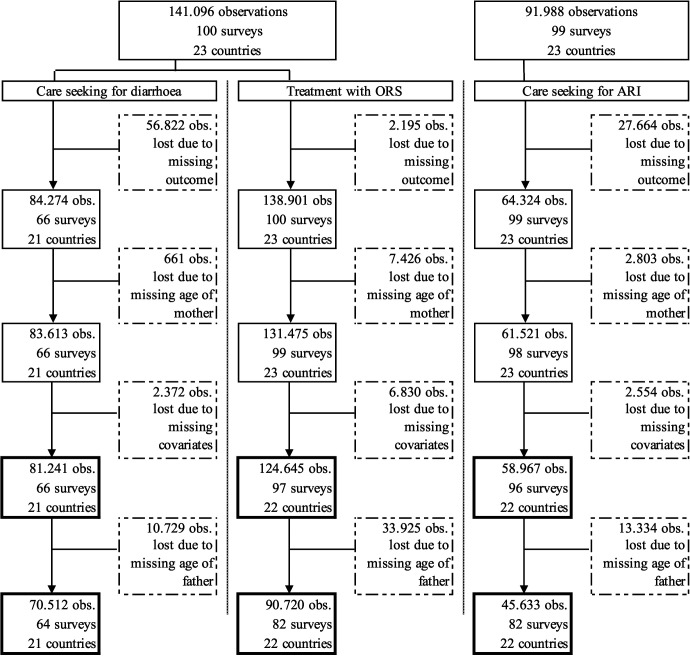
Sample selection.

In this analysis, we included surveys from 1995 to 2017 and all countries in Western and Central Africa for which data on outcomes, parents’ age, and covariates was available. Observations with missing information in any of these variables had to be excluded from the analysis. **Figure 1** presents the sample selection in greater detail. The regional focus was motivated by the fact that several of the countries with the highest burden of diarrhoea and pneumonia are located in Western and Central Africa, including Nigeria, the Democratic Republic of Congo, Chad, Niger, and Côte d’Ivoire [3] and progress in reducing the disease burden has been slowing down in the past decades [4].

Our sample included only children, 0 to 59 months of age, that were affected by diarrhoea or ARI, respectively. A child was defined as having diarrhoea or fever if his/her guardian responded that the child suffered from diarrhoea or fever in the past 24 hours. A child was defined as having ARI if he/she had cough in the past 24 hours and experienced short, rapid breathing.

### Outcomes

We analysed the association of mother’s and father’s age at birth of the child with care seeking behaviour for the two childhood diseases diarrhoea and ARI. The outcome variables were a) whether care was sought anywhere outside home for a child who was sick with diarrhoea (*care seeking for diarrhoea)*, b) whether care was sought anywhere outside home for a child who was sick with ARI (*care seeking for ARI*), and c) whether a child who was sick with diarrhoea was treated with ORS (*treatment with ORS*). The latter outcome was included based on the recommendation by WHO and UNICEF to treat acute diarrhoea with ORS [15]. All outcome variables were coded as dummy variables that equal one if the respective care was given to the sick child. *Treatment with ORS* was captured in a different survey question than *care seeking for diarrhoea*. Not all surveys include both questions. The outcome *treatment with ORS* includes ORS given at a health facility and ORS given by parents. However, there is correlation between the two outcomes. While 44 percent of children for whom care was sought outside home for diarrhoea were also given ORS as treatment, only 11 percent of children for whom no care was sought outside home for diarrhoea received ORS.

### Exposure and covariates

The main variables of interest in this analysis were the age of the child’s mother and father at birth of the respective child. As the available data does not identify the true father of the child, we used the age of the mother’s husband as a proxy, assuming that he would fulfil the role of a father in most cases. We created 5-year age groups for the mother (<20, 20-24, 25-29, 30-34, 35-39, 40+ years) and the father (<25, 25-29, 30-34, 35-39, 40-44, 45+ years) to form categorical age variables.

Covariates used in the analysis included the child’s gender and age in years, the mother’s educational level (none, primary, or secondary and higher), the location of the household (rural or urban), as well as the wealth quartile of the household. Educational level and wealth were included as socioeconomic characteristics as they have been shown to be associated with adolescent maternity [16]. The wealth quartile was based on an asset index of observed household assets. Our asset index was calculated using principal-component analysis to define asset-based survey-specific wealth quartiles based on the same list of assets for each survey. Included assets were radio, television, bicycle, car, motorcycle, refrigerator, phone, electricity, piped drinking water, flush toilet, floor material, wall material, and roof material. Households with information on less than 88% of underlying assets were excluded from the calculation of the asset index. This cut-off was chosen to include the maximum information about assets while retaining as many country-years as possible.

### Statistical analysis

We estimated the association of mother’s and father’s age at birth with care seeking behaviour in three models. First, we looked at the simple association between care seeking behaviour and parents’ age. To avoid the assumption of a linear relationship, age was included as categorical variable. The two middle age groups formed the reference group for the estimations, 25-34 years for the mother and 30-39 years for the father. Coefficients should be interpreted as the difference in probability of care seeking compared to the probability if the mother or father belongs to the reference group.

In a second step, we added covariates capturing child characteristics including gender and age, mother’s education, location, as well as the socioeconomic status of the child’s household. Furthermore, we added survey year- and country-fixed effects as country level factors have been shown to influence childhood diseases such as diarrhoea [17]. The third model included PSU fixed effects.

As information about the father is not available for all observations, we first estimated the three models including only the mother’s age (columns 1-3 in **Tables 1 **to** 3**). Next, we estimated model 3 which includes the mother’s age, covariates, as well as PSU fixed effects for the subset of observations that also contain information about the father (column 4). Lastly, we estimated the three models including both the mother’s and the father’s age (columns 5-7).

**Table 1:** Regression results for outcome Care seeking for diarrhoea*

	(1)	(2)	(3)	(4)	(5)	(6)	(7)
Mother's age at birth: <20	-0.0005 (0.0125)	0.0008 (0.0135)	0.0047 (0.0138)	0.0027 (0.0159)	-0.0137 (0.0171)	-0.0007 (0.0172)	0.0044 (0.0169)
Mother's age at birth: 20-24	-0.0028 (0.0099)	0.0008 (0.0105)	-0.0039 (0.0112)	-0.0049 (0.0125)	-0.0099 (0.0123)	-0.0000 (0.0121)	-0.0044 (0.0129)
Mother's age at birth: 35-39	-0.0179 (0.0134)	-0.0146 (0.0132)	-0.0195 (0.0147)	-0.0253 (0.0162)	0.0044 (0.0149)	-0.0012 (0.0143)	-0.0163 (0.0160)
Mother's age at birth: 40+	-0.0370† (0.0189)	-0.0162 (0.0204)	0.0048 (0.0200)	0.0033 (0.0213)	-0.0096 (0.0210)	-0.0024 (0.0223)	0.0088 (0.0217)
Father's age at birth: <25					-0.0162 (0.0187)	-0.0200 (0.0193)	-0.0135 (0.0213)
Father's age at birth: 25-29					-0.0046 (0.0141)	-0.0082 (0.0141)	-0.0130 (0.0144)
Father's age at birth: 40-44					-0.0200 (0.0139)	-0.0184 (0.0136)	-0.0167 (0.0157)
Father's age at birth: 44+					-0.0368§ (0.0134)	-0.0241† (0.0136)	-0.0193 (0.0144)
Gender of child: female		-0.0150† (0.0077)	-0.0184‡ (0.0091)	-0.0186† (0.0101)		-0.0173‡ (0.0078)	-0.0199‡ (0.0095)
Age of child: 1 y		0.0250§ (0.0097)	0.0227‡ (0.0115)	0.0219† (0.0126)		0.0313§ (0.0101)	0.0287‡ (0.0120)
Age of child: 2 y		0.0198† (0.0111)	0.0096 (0.0129)	0.0168 (0.0142)		0.0265‡ (0.0114)	0.0185 (0.0134)
Age of child: 3 y		-0.0076 (0.0128)	-0.0110 (0.0153)	-0.0047 (0.0168)		-0.0010 (0.0133)	0.0001 (0.0158)
Age of child: 4 y		-0.0190 (0.0169)	-0.0047 (0.0183)	0.0099 (0.0206)		-0.0073 (0.0174)	0.0107 (0.0195)
Mother with primary education		0.0408§ (0.0115)	0.0322‡ (0.0130)	0.0382§ (0.0144)		0.0480§ (0.0117)	0.0366§ (0.0136)
Mother with secondary education +		0.0485§ (0.0136)	0.0653§ (0.0180)	0.0667§ (0.0211)		0.0439§ (0.0147)	0.0640§ (0.0200)
Household in rural area		0.0096 (0.0142)				-0.0002 (0.0146)	
Household in 2nd wealth quartile		0.0310‡ (0.0121)	0.0207 (0.0138)	0.0197 (0.0154)		0.0309‡ (0.0125)	0.0208 (0.0145)
Household in 3rd wealth quartile		0.0624§ (0.0153)	0.0345‡ (0.0159)	0.0276 (0.0179)		0.0566§ (0.0155)	0.0270 (0.0169)
Household in 4th wealth quartile		0.0685§ (0.0175)	0.0539§ (0.0206)	0.0397† (0.0229)		0.0584§ (0.0180)	0.0386† (0.0216)
PSU fixed effects	No	No	Yes	Yes	No	No	Yes
Number of countries	21	21	21	21	21	21	21
Number of surveys	66	66	66	64	64	64	64

PSU – primary sampling unit, y – year

*Regression results for outcome *Care seeking for diarrhoea.* Reference categories are age group 25-34 for mother’s age, age group 30-39 for father’s age, children below 1 year for child’s age, mothers with no education for mother’s education, poorest wealth quartile for wealth status. All models include survey-year and country-fixed effects. Columns (3) and (7) additionally include PSU fixed effects. Columns (1) to (3) include the sample with observations for mother’s age. Columns (4) to (7) include the sample with observations for mother’s and father’s age. Standard errors in parentheses.

†*P* < 0.1.

‡*P* < 0.05.

§*P* < 0.01.

**Table 2:** Regression results for outcome Care seeking for ARI*

	(1)	(2)	(3)	(4)	(5)	(6)	(7)
Mother's age at birth: <20	-0.0075 (0.0134)	-0.0088 (0.0142)	-0.0122 (0.0177)	-0.0186 (0.0234)	-0.0034 (0.0180)	-0.0100 (0.0174)	-0.0369 (0.0231)
Mother's age at birth: 20-24	-0.0005 (0.0104)	-0.0055 (0.0108)	-0.0045 (0.0134)	0.0013 (0.0160)	0.0004 (0.0130)	-0.0072 (0.0130)	-0.0123 (0.0175)
Mother's age at birth: 35-39	-0.0177 (0.0130)	-0.0028 (0.0135)	-0.0124 (0.0179)	-0.0137 (0.0196)	-0.0037 (0.0168)	0.0071 (0.0173)	-0.0043 (0.0200)
Mother's age at birth: 40+	-0.0193 (0.0187)	0.0105 (0.0197)	-0.0030 (0.0247)	-0.0122 (0.0316)	-0.0020 (0.0244)	0.0174 (0.0251)	0.0134 (0.0329)
Father's age at birth: <25					-0.0405† (0.0222)	-0.0174 (0.0229)	0.0263 (0.0328)
Father's age at birth: 25-29					-0.0195 (0.0148)	-0.0051 (0.0143)	0.0028 (0.0188)
Father's age at birth: 40-44					-0.0167 (0.0158)	-0.0233 (0.0168)	0.0129 (0.0201)
Father's age at birth: 44+					-0.0232 (0.0170)	-0.0141 (0.0170)	-0.0382† (0.0197)
Gender of child: female		-0.0089 (0.0089)	-0.0196† (0.0108)	-0.0203 (0.0130)		-0.0143 (0.0101)	-0.0212† (0.0129)
Age of child: 1 y		0.0118 (0.0127)	0.0205 (0.0151)	0.0117 (0.0179)		0.0040 (0.0144)	0.0152 (0.0176)
Age of child: 2 y		-0.0019 (0.0119)	-0.0030 (0.0151)	0.0022 (0.0185)		-0.0083 (0.0136)	0.0042 (0.0187)
Age of child: 3 y		-0.0418§ (0.0147)	-0.0346‡ (0.0168)	-0.0274 (0.0205)		-0.0419‡ (0.0173)	-0.0261 (0.0201)
Age of child: 4 y		-0.0331‡ (0.0139)	-0.0177 (0.0184)	-0.0179 (0.0197)		-0.0370‡ (0.0162)	-0.0138 (0.0203)
Mother with primary education		0.0507§ (0.0120)	0.0350‡ (0.0153)	0.0377‡ (0.0184)		0.0568§ (0.0137)	0.0313† (0.0184)
Mother with secondary education +		0.0816§ (0.0144)	0.0839§ (0.0213)	0.0915§ (0.0269)		0.0943§ (0.0156)	0.0867§ (0.0269)
Household in rural area		-0.0493§ (0.0127)				-0.0482§ (0.0141)	
Household in 2nd wealth quartile		0.0508§ (0.0132)	0.0362† (0.0191)	0.0332 (0.0227)		0.0408§ (0.0138)	0.0261 (0.0221)
Household in 3rd wealth quartile		0.0707§ (0.0128)	0.0261 (0.0197)	0.0241 (0.0241)		0.0628§ (0.0140)	0.0197 (0.0238)
Household in 4th wealth quartile		0.1169§ (0.0155)	0.0548‡ (0.0242)	0.0496† (0.0287)		0.1078§ (0.0166)	0.0447 (0.0277)
PSU fixed effects	No	No	Yes	Yes	No	No	Yes
Number of countries	23	22	22	22	22	22	22
Number of surveys	98	96	96	82	83	82	82

ARI – acute respiratory infection, PSU – primary sampling unit, y – year

*Regression results for outcome *Care seeking for ARI.* Reference categories are age group 25-34 for mother’s age, age group 30-39 for father’s age, children below 1 year for child’s age, mothers with no education for mother’s education, poorest wealth quartile for wealth status. All models include survey-year and country-fixed effects. Columns (3) and (7) additionally include PSU fixed effects. Columns (1) to (3) include the sample with observations for mother’s age. Columns (4) to (7) include the sample with observations for mother’s and father’s age. Standard errors in parentheses.

†*P* < 0.1.

‡*P* < 0.05.

§*P* < 0.01.

**Table 3:** Regression results for outcome variable Treatment with ORS*

	(1)	(2)	(3)	(4)	(5)	(6)	(7)
Mother's age at birth: <20	-0.0297§ (0.0071)	-0.0290§ (0.0077)	-0.0152† (0.0086)	-0.0105 (0.0119)	-0.0524§ (0.0109)	-0.0344§ (0.0116)	-0.0193 (0.0133)
Mother's age at birth: 20-24	-0.0123‡ (0.0058)	-0.0151‡ (0.0065)	-0.0071 (0.0067)	-0.0134 (0.0087)	-0.0307§ (0.0078)	-0.0210‡ (0.0083)	-0.0157† (0.0092)
Mother's age at birth: 35-39	-0.0192‡ (0.0083)	-0.0129 (0.0086)	-0.0098 (0.0096)	-0.0165 (0.0124)	-0.0138 (0.0105)	-0.0144 (0.0104)	-0.0151 (0.0121)
Mother's age at birth: 40+	-0.0312§ (0.0106)	-0.0071 (0.0121)	-0.0100 (0.0122)	-0.0129 (0.0165)	-0.0159 (0.0144)	-0.0089 (0.0156)	-0.0133 (0.0172)
Father's age at birth: <25					0.0132 (0.0131)	0.0060 (0.0139)	0.0196 (0.0154)
Father's age at birth: 25-29					0.0075 (0.0092)	0.0038 (0.0096)	0.0061 (0.0107)
Father's age at birth: 40-44					-0.0145 (0.0097)	-0.0099 (0.0104)	-0.0046 (0.0113)
Father's age at birth: 44+					-0.0227‡ (0.0092)	0.0010 (0.0101)	0.0016 (0.0101)
Gender of child: female		-0.0057 (0.0051)	-0.0071 (0.0056)	-0.0117 (0.0074)		-0.0099† (0.0059)	-0.0134† (0.0070)
Age of child: 1 year		0.0310§ (0.0068)	0.0325§ (0.0070)	0.0366§ (0.0090)		0.0367§ (0.0079)	0.0383§ (0.0086)
Age of child: 2 year		0.0027 (0.0082)	0.0114 (0.0082)	0.0143 (0.0107)		0.0036 (0.0093)	0.0134 (0.0101)
Age of child: 3 year		-0.0270§ (0.0080)	-0.0151† (0.0088)	-0.0075 (0.0116)		-0.0240‡ (0.0095)	-0.0040 (0.0112)
Age of child: 4 year		-0.0060 (0.0096)	0.0096 (0.0095)	0.0138 (0.0125)		-0.0058 (0.0112)	0.0114 (0.0118)
Mother with primary education		0.0419§ (0.0074)	0.0269§ (0.0083)	0.0327§ (0.0113)		0.0428§ (0.0088)	0.0348§ (0.0105)
Mother with secondary education +		0.0936§ (0.0098)	0.0791§ (0.0125)	0.0960§ (0.0173)		0.0944§ (0.0117)	0.0948§ (0.0164)
Household in rural area		-0.0320§ (0.0080)				-0.0243‡ (0.0095)	
Household in 2^nd^ wealth quartile		0.0325§ (0.0075)	0.0261§ (0.0086)	0.0287§ (0.0109)		0.0407§ (0.0086)	0.0302§ (0.0103)
Household in 3^rd^ wealth quartile		0.0555§ (0.0082)	0.0382§ (0.0101)	0.0369§ (0.0138)		0.0682§ (0.0100)	0.0370§ (0.0130)
Household in 4^th^ wealth quartile		0.1046§ (0.0106)	0.0920§ (0.0135)	0.0832§ (0.0179)		0.1140§ (0.0125)	0.0789§ (0.0166)
PSU fixed effects	No	No	Yes	Yes	No	No	Yes
Number of countries	23	22	22	22	22	22	22
Number of surveys	99	97	97	82	83	82	82

ORS – oral rehydration solution, PSU – primary sampling unit, y – year

*Regression results for outcome *Treatment with ORS in case of diarrhoea.* Reference categories are age group 25-34 for mother’s age, age group 30-39 for father’s age, children below 1 year for child’s age, mothers with no education for mother’s education, poorest wealth quartile for wealth status. All models include survey-year and country-fixed effects. Columns (3) and (7) additionally include PSU fixed effects. Columns (1) to (3) include the sample with observations for mother’s age. Columns (4) to (7) include the sample with observations for mother’s and father’s age. Standard errors in parentheses.

†*P* < 0.1.

‡*P* < 0.05.

§*P* < 0.01.

In addition, we analysed the association between health care seeking and parents’ age for three different time periods (**Tables 4 **to** 6**). Time periods were defined such that each country had at least one survey in each time period, as far as possible. This resulted in the earliest period containing surveys between 1995 and 2001, the middle period containing surveys between 2002 and 2009, and the most recent time period containing surveys between 2010 and 2017.

**Table 4:** Regression results over time for outcome variable Care seeking for diarrhoea*

	(1)	(2)	(3)	(4)	(5)	(6)	(7)
**Period 1995-2001:**							
Mother's age at birth: <20	-0.0643‡ (0.0257)	-0.0651‡ (0.0257)	-0.0585‡ (0.0274)	-0.0707‡ (0.0317)	-0.1055§ (0.0361)	-0.1025§ (0.0350)	-0.1147§ (0.0383)
Mother's age at birth: 40+	-0.0573 (0.0430)	-0.0263 (0.0405)	-0.0222 (0.0418)	0.0020 (0.0481)	0.0113 (0.0511)	-0.0127 (0.0537)	0.0311 (0.0504)
Father's age at birth: <25					0.0682 (0.0449)	0.0585 (0.0456)	0.0914‡ (0.0454)
Father's age at birth: 45+					-0.0343 (0.0316)	-0.0403 (0.0304)	-0.0524† (0.0308)
Number of countries	13	13	13	11	11	11	11
Number of surveys	15	15	15	13	13	13	13
**Period 2002-2009:**							
Mother's age at birth: <20	0.0210 (0.0230)	0.0266 (0.0229)	0.0228 (0.0229)	0.0196 (0.0249)	0.0304 (0.0297)	0.0162 (0.0302)	0.0214 (0.0279)
Mother's age at birth: 40+	0.0086 (0.0297)	0.0223 (0.0309)	0.0368 (0.0293)	0.0321 (0.0301)	0.0416 (0.0363)	0.0350 (0.0342)	0.0428 (0.0331)
Father's age at birth: <25					-0.0260 (0.0328)	-0.0217 (0.0324)	-0.0051 (0.0360)
Father's age at birth: 45+					-0.0219 (0.0230)	-0.0357 (0.0228)	-0.0199 (0.0241)
Number of countries	15	15	15	15	15	15	15
Number of surveys	17	17	17	17	17	17	17
**Period 2010-2017:**							
Mother's age at birth: <20	0.0172 (0.0134)	0.0090 (0.0139)	0.0148 (0.0146)	0.0228 (0.0167)	0.0150 (0.0171)	0.0245 (0.0167)	0.0373† (0.0191)
Mother's age at birth: 40+	-0.0553‡ (0.0244)	-0.0500‡ (0.0250)	-0.0246 (0.0271)	-0.0336 (0.0291)	-0.0484† (0.0253)	-0.0448† (0.0251)	-0.0305 (0.0305)
Father's age at birth: <25					-0.0326 (0.0217)	-0.0378† (0.0218)	-0.0476† (0.0244)
Father's age at birth: 45+					-0.0146 (0.0150)	-0.0239 (0.0147)	-0.0075 (0.0174)
Number of countries	21	21	21	21	21	21	21
Number of surveys	34	34	34	34	34	34	34
Control variables	No	Yes	Yes	Yes	No	Yes	Yes
PSU fixed effects	No	No	Yes	Yes	No	No	Yes

PSU – primary sampling unit

*Regression results for outcome *Care seeking for diarrhoea,* by time period. Reference categories are age group 25-34 for mother’s age, age group 30-39 for father’s age, children below 1 year for child’s age, mothers with no education for mother’s education, poorest wealth quartile for wealth status. All models include survey-year and country-fixed effects. Columns (3) and (7) additionally include PSU fixed effects. Columns (1) to (3) include the sample with observations for mother’s age. Columns (4) to (7) include the sample with observations for mother’s and father’s age. Standard errors in parentheses.

†*P* < 0.1.

‡*P* < 0.05.

§*P* < 0.01.

**Table 5:** Regression results over time for outcome variable *Care seeking for ARI*

	(1)	(2)	(3)	(4)	(5)	(6)	(7)
**Period 1995-2001:**							
Mother's age at birth: <20	-0.0067 (0.0277)	-0.0103 (0.0284)	0.0146 (0.0316)	0.0007 (0.0564)	-0.0407 (0.0464)	-0.0529 (0.0454)	-0.0133 (0.0536)
Mother's age at birth: 40+	-0.0184 (0.0348)	0.0222 (0.0343)	-0.0014 (0.0349)	0.0068 (0.0611)	0.0069 (0.0531)	-0.0229 (0.0515)	0.0077 (0.0569)
Father's age at birth: <25					0.0368 (0.0568)	-0.0017 (0.0554)	0.0500 (0.0787)
Father's age at birth: 45+					0.0113 (0.0298)	-0.0244 (0.0305)	-0.0102 (0.0319)
Number of countries	20	19	19	11	11	11	11
Number of surveys	27	26	26	13	13	13	13
**Period 2002-2009:**							
Mother's age at birth: <20	-0.0331 (0.0278)	-0.0285 (0.0282)	-0.0299 (0.0378)	-0.0194 (0.0429)	-0.0225 (0.0288)	-0.0127 (0.0300)	-0.0254 (0.0429)
Mother's age at birth: 40+	-0.0125 (0.0410)	0.0145 (0.0410)	0.0126 (0.0546)	0.0023 (0.0608)	0.0322 (0.0490)	0.0097 (0.0496)	0.0513 (0.0628)
Father's age at birth: <25					-0.0269 (0.0432)	-0.0515 (0.0393)	-0.0051 (0.0660)
Father's age at birth: 45+					-0.0310 (0.0329)	-0.0293 (0.0352)	-0.0758‡ (0.0373)
Number of countries	21	21	21	20	20	20	20
Number of surveys	28	27	27	26	27	26	26
**Period 2010-2017:**							
Mother's age at birth: <20	-0.0007 (0.0187)	0.0079 (0.0192)	-0.0280 (0.0195)	-0.0338 (0.0215)	0.0203 (0.0240)	0.0051 (0.0239)	-0.0591‡ (0.0247)
Mother's age at birth: 40+	0.0061 (0.0272)	0.0193 (0.0284)	-0.0081 (0.0312)	-0.0207 (0.0345)	0.0187 (0.0331)	0.0158 (0.0321)	-0.0123 (0.0371)
Father's age at birth: <25					-0.0198 (0.0272)	-0.0294 (0.0278)	0.0532† (0.0298)
Father's age at birth: 45+					-0.0005 (0.0215)	-0.0193 (0.0210)	-0.0046 (0.0233)
Number of countries	22	22	22	22	22	22	22
Number of surveys	43	43	43	43	43	43	43
Control variables	No	Yes	Yes	Yes	No	Yes	Yes
PSU fixed effects	No	No	Yes	Yes	No	No	Yes

ARI – acute respiratory infection, PSU – primary sampling unit

*Regression results for outcome *Care seeking for ARI*, by time period. Reference categories are age group 25-34 for mother’s age, age group 30-39 for father’s age, children below 1 year for child’s age, mothers with no education for mother’s education, poorest wealth quartile for wealth status. All models include survey-year and country-fixed effects. Columns (3) and (7) additionally include PSU fixed effects. Columns (1) to (3) include the sample with observations for mother’s age. Columns (4) to (7) include the sample with observations for mother’s and father’s age. Standard errors in parentheses.

†*P* < 0.1.

‡*P* < 0.05.

§*P* < 0.01.

**Table 6:** Regression results over time for outcome variable Treatment with ORS*

	(1)	(2)	(3)	(4)	(5)	(6)	(7)
**Period 1995 – 2001:**							
Mother's age at birth: <20	-0.0315† (0.0161)	-0.0387‡ (0.0152)	-0.0121 (0.0135)	-0.0238 (0.0296)	-0.0922§ (0.0347)	-0.0921‡ (0.0361)	-0.0619† (0.0325)
Mother's age at birth: 40+	-0.0500‡ (0.0218)	-0.0114 (0.0213)	-0.0180 (0.0198)	-0.0147 (0.0501)	0.0174 (0.0484)	-0.0181 (0.0476)	-0.0035 (0.0462)
Father's age at birth: <25					0.0315 (0.0473)	0.0203 (0.0466)	0.0764† (0.0424)
Father's age at birth: 45+					-0.0295 (0.0303)	-0.0420 (0.0296)	-0.0182 (0.0291)
Number of countries	20	19	19	11	11	11	11
Number of surveys	28	27	27	13	13	13	13
**Period 2002-2009:**							
Mother's age at birth: <20	-0.0364§ (0.0141)	-0.0190 (0.0144)	-0.0174 (0.0177)	-0.0104 (0.0212)	-0.0069 (0.0212)	-0.0478‡ (0.0193)	-0.0098 (0.0257)
Mother's age at birth: 40+	-0.0052 (0.0193)	0.0176 (0.0211)	0.0111 (0.0266)	0.0067 (0.0322)	-0.0064 (0.0256)	-0.0038 (0.0233)	-0.0094 (0.0334)
Father's age at birth: <25					-0.0093 (0.0246)	0.0109 (0.0236)	0.0194 (0.0297)
Father's age at birth: 45+					0.0272 (0.0179)	-0.0108 (0.0154)	0.0263 (0.0181)
Number of countries	21	21	21	20	20	20	20
Number of surveys	28	27	27	26	27	26	26
**Period 2010-2017:**							
Mother's age at birth: <20	-0.0304§ (0.0111)	-0.0318§ (0.0111)	-0.0072 (0.0113)	0.0081 (0.0129)	-0.0355‡ (0.0144)	-0.0391§ (0.0147)	-0.0047 (0.0142)
Mother's age at birth: 40+	-0.0469‡ (0.0200)	-0.0161 (0.0198)	-0.0170 (0.0172)	-0.0224 (0.0192)	-0.0053 (0.0236)	-0.0304 (0.0234)	-0.0098 (0.0207)
Father's age at birth: <25					0.0114 (0.0188)	0.0061 (0.0188)	0.0103 (0.0186)
Father's age at birth: 45+					-0.0158 (0.0141)	-0.0257† (0.0138)	-0.0186 (0.0128)
Number of countries	22	22	22	22	22	22	22
Number of surveys	43	43	43	43	43	43	43
Control variables	No	Yes	Yes	Yes	No	Yes	Yes
PSU fixed effects	No	No	Yes	Yes	No	No	Yes

ORS – oral rehydration solution, PSU – primary sampling unit.

*Regression results for outcome *Treatment with ORS in case of diarrhoea*, by time period. Reference categories are age group 25-34 for mother’s age, age group 30-39 for father’s age, children below 1 year for child’s age, mothers with no education for mother’s education, poorest wealth quartile for wealth status. All models include survey-year and country-fixed effects. Columns (3) and (7) additionally include PSU fixed effects. Columns (1) to (3) include the sample with observations for mother’s age. Columns (4) to (7) include the sample with observations for mother’s and father’s age. Standard errors in parentheses.

†*P* < 0.1.

‡*P* < 0.05.

§*P* < 0.01.

To investigate heterogeneities in the association between countries, model 3 including father’s age (column 7) was re-estimated for each country separately, both across time and for different time periods. A list of included countries and abbreviations for country names used in figures is shown in **Table 7**.

**Table 7:** List of countries

Country name	Country code
Burkina Faso	BF
Benin	BJ
Congo Democratic Republic	CD
Central African Republic	CF
Congo	CG
Cote d'Ivoire	CI
Cameroon	CM
Equatorial Guinea	EQG
Gabon	GA
Ghana	GH
Gambia	GM
Guinea	GN
Guinea-Bissau	GW
Liberia	LB
Mauritania	MAU
Mali	ML
Nigeria	NG
Niger	NI
Sierra Leone	SL
Senegal	SN
São Tomé and Príncipe	ST
Chad	TD
Togo	TG

We did not adjust for multiple hypotheses testing. All analyses were performed using Stata 14.

### Ethical approval

Ethical approval was not obtained for this study due to the use of secondary data. Both DHS and MICS obtain ethical approval for data collection.

## RESULTS

The final sample for the analysis with covariates included 81 241 observations from 66 surveys and 21 countries for the outcome *care seeking for diarrhoea*, 124 645 observations from 97 surveys and 22 countries for the outcome *treatment with ORS*, and 58 967 observations from 96 surveys and 22 countries for the outcome *care seeking for ARI*. The differences resulted from the different prevalence of diarrhoea and ARI as well as the fact that more surveys included questions about care seeking for ARI than for diarrhoea. Including father’s age in the analysis further reduced the sample. Details on sample selection are shown in **Figure 1**.

**Figure 2:**
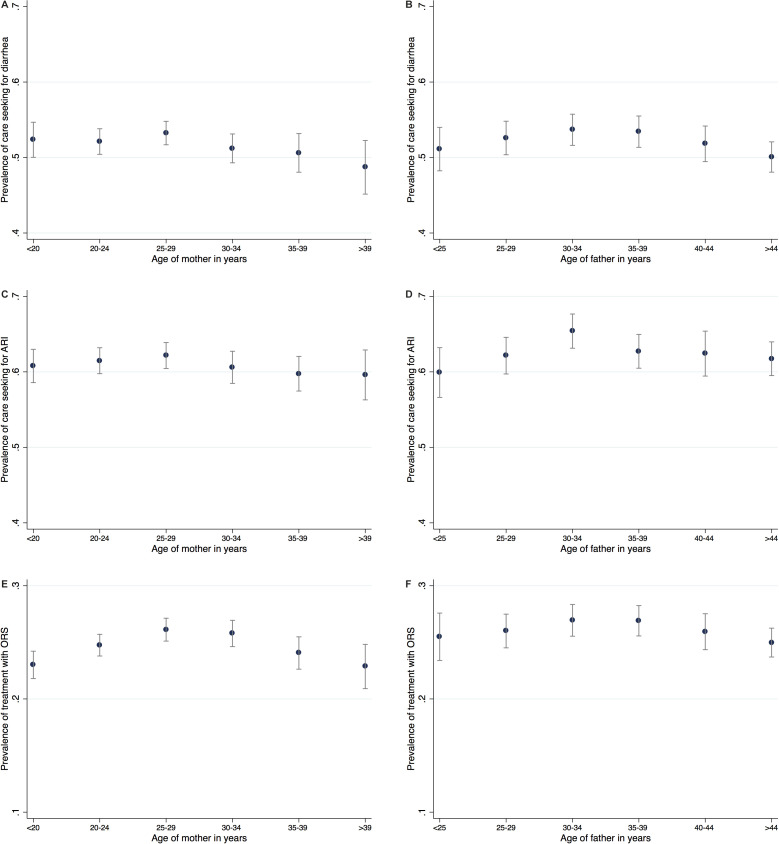
Prevalence of care seeking across age groups of parents. Panel A. Prevalence of Care seeking for diarrhoea across age groups of mother. Panel B. Prevalence of Care seeking for diarrhoea across age groups of father. Panel C. Prevalence of Care seeking for ARI across age groups of mother. Panel D. Prevalence of Care seeking for ARI across age groups of father. Panel E. Prevalence of Treatment with ORS across age groups of mother. Panel F. Prevalence of Treatment with ORS across age groups of father. Note: Prevalence of care seeking by age group of mother and father, across all countries, weighted by each country’s female population aged 15 to 49 years in 2017, means and 95% confidence intervals. ARI – acute respiratory infections. ORS – oral rehydration solution.

**Figure 2** (panels A-D) depict the likelihood that care is sought from any source for a child with diarrhoea or ARI for different age groups of the mother and the father. The graphs suggest that mothers and fathers in the middle age categories are slightly more likely to seek care for their child. However, the differences between age groups do not seem to be statistically significant as confidence intervals overlap for all age groups. The likelihood that care is sought for a child with diarrhoea ranges between 48.7% (95% confidence interval (CI) = 45.1%-52.3%) among mothers above 40 years to 53% (95% CI = 51.7%-54.8%) among mothers between 25 and 29 years. The pattern is similar for ARI, but with higher likelihoods for all age groups. The likelihood that care is sought for a child with ARI ranges between 59.6% (95% CI = 56.3%-62.3%) for the oldest age group to 62.2% (95% CI = 60.4%-63.9%) for mothers aged 25 to 29 years.

**Table 1** and **Table 2** show the results of pooled regressions across all included countries between 1995 and 2017 for the outcomes *care seeking for diarrhoea* and *care seeking for ARI*. Our main specification which includes both parents’ age, covariates, as well as PSU fixed effects (column 7) shows no significant association of parents’ age with care seeking in case the child has diarrhoea. The coefficients are small and insignificant. The pattern largely persists for our alternative specification without PSU fixed effects (column 6) or without father’s age (columns 2 and 3). Only when looking at the most basic models that include only the age of the mother or of both parents and no further controls (columns 1 and 5) some of the coefficients are significantly different from zero. In this basic model, mothers who were 40 years or older at birth have a 3.7 percentage point lower likelihood of seeking care for their child with diarrhoea. When father’s age is included, we find a corresponding 3.7 percentage point lower likelihood for children with fathers in the oldest age group, but the association for the oldest mothers disappears. The associations are similarly weak for seeking care in case the child has ARI. No coefficient for age of mother at child’s birth is statistically significant in any specification. A weakly significant association is found again for children with fathers in the oldest age group in the main specification including PSU fixed effects, suggesting a lower probability of 3.8 percentage points.

We find strong positive associations of the level of education of the mother. Mothers with at least primary education are 3.7 percentage points more likely than mothers without formal education to seek care for their child when he/she has diarrhoea and 3.1 percentage points more likely when the child has ARI. Coefficients for wealth are significant only without PSU fixed effects. In model 2 with father’s age (column 6) compared to children from the poorest quartile, children from the second wealth quartile are 3.1 percentage points more likely to be taken anywhere for care seeking when having diarrhoea and children from the richest quartile are almost 6 percentage points more likely to be taken anywhere for care seeking. While coefficients remain sizable, they turn insignificant once we control for PSU. In case of ARI the differences increase to 4.1 percentage points for children from the second quartile and 10.8 percentage points for children from the richest quartile. The location of the household cannot be included in models with PSU fixed effects as a PSU is either rural or urban. In model 2 (column 6), whether the household is located in a rural or urban area is not associated with care seeking behaviour for diarrhoea but in case the child suffers from ARI, he/she is 4.8 percentage points less likely to be taken anywhere for care seeking when the household lives in a rural area.

Care seeking seems to be highest for children at one year of age and decreasing as they grow older. Parents of children aged between one and two years have a 2.9 percentage points higher probability of seeking care for diarrhoea than those of children below one year of age (column 7). Children aged three or four years have a lower likelihood of being taken for care for ARI compared to children below one year of age. However, this association is only significant without PSU fixed effects. In case of diarrhoea and ARI, girls are 2.0 and 2.1 percentage points less likely to be taken anywhere for care, respectively (column 7).

**Figure 3:**
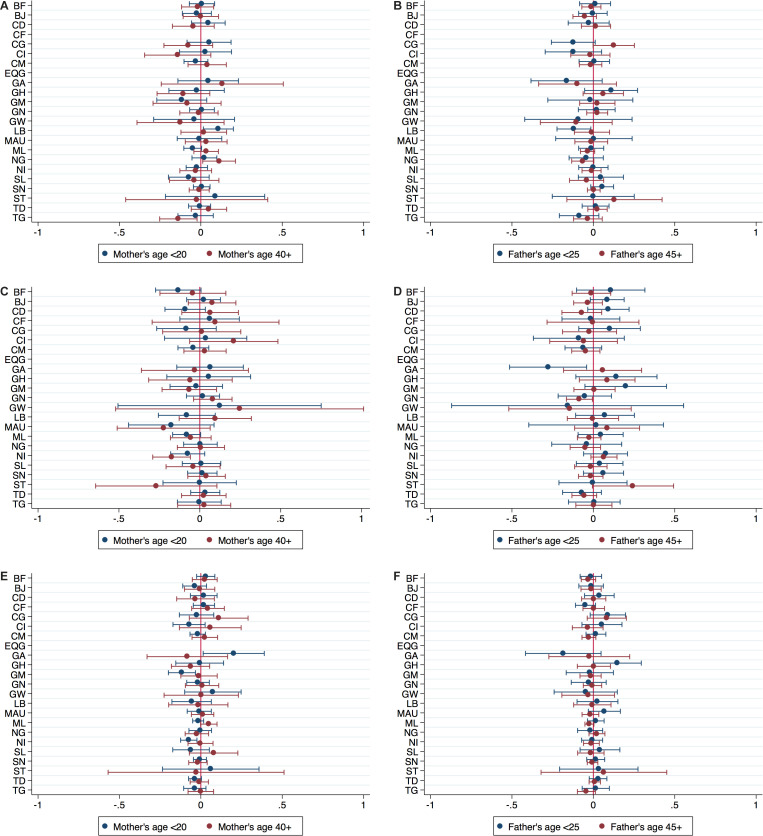
Coefficients of parents’ age from model 3 (with father’s age) by country. Panel A. Coefficients of mother’s age, outcome variable: Care seeking for diarrhoea. Panel B. Coefficients of father’s age, outcome variable: Care seeking for diarrhoea. Panel C. Coefficients of mother’s age, outcome variable: Care seeking for ARI. Panel D. Coefficients of father’s age, outcome variable: Care seeking for ARI. Panel E. Coefficients of mother’s age, outcome variable: Treatment with ORS. Panel F. Coefficients of father’s age, outcome variable: Treatment with ORS. Note: Coefficients and confidence intervals of mother’s age, age categories <20 and 40+, and father’s age, age categories <25 and 45+, based on country-specific regressions following column (7) in **Table 1**, **Table 2**, **Table 3**, respectively. ARI – acute respiratory infections. ORS – oral rehydration solution.

Looking at individual countries yields a similar picture. **Figure 3** (panels A-D) present the coefficients of the main variables of interest, mother’s and father’s age taken from country-specific regressions that include all covariates and PSU fixed effects (corresponding to column 7). They depict the coefficients of the respective youngest and oldest age group, the reference group being 25-34 years for mothers and 30-39 years for fathers. Estimations were not possible for Equatorial Guinea for either outcome and Central African Republic for *care seeking for diarrhoea* due to missing information on outcome variables. For *care seeking for diarrhoea,* coefficients of mother’s and father’s age are close to zero and statistically insignificant for most countries, with few exceptions. Mothers in the youngest age group seem to be more likely, while fathers in the youngest age group seem to be less likely to seek care for diarrhoea in Liberia compared to the reference categories. Mothers in the oldest age group seem to be more likely to seek care in Nigeria, but less likely in Togo. For *care seeking for ARI,* findings are similarly inconsistent and mostly insignificant. In Niger, the probability of care seeking is lower for children of mothers above 40 years. The probability of care seeking is also lower for children of fathers below 25 years in Gabon and for children of fathers above 45 years in Guinea.

The third outcome analysed is treatment with ORS in case of diarrhoea. The likelihood of ORS treatment across all included countries as shown in **Figure 2** (panels E-F) ranges from 22.8 percent (95% CI = 20.9%-24.8%) for mothers older than 40 years to 26.1 percent (95% CI = 25.1%-27.1%) for mothers between 25 and 29 years. Similarly, the likelihood is lowest for fathers older than 45 years and highest for fathers between 30 and 39 years. These graphs suggest a slightly more pronounced pattern of an inverse U-shape than the graphs for the other care seeking outcomes.

The most basic model (column 1 in **Table 3**) confirms the inverse U-shape for the association between treatment with ORS and mother’s age, suggesting lower treatment probability for children of mothers in the youngest and oldest age group. While the sign of coefficients stays the same across specifications, the relationship weakens when covariates and PSU fixed effects are included. The main specification of our analysis (column 7) shows an inverse U-shape pattern for mother’s age, with a weakly significant negative coefficient for age group 20-24 years. However, coefficients for the other age groups and for father’s age are statistically insignificant. Patterns of the association with mother’s education and wealth status are similar to the analysis for the other two care seeking outcomes and both characteristics remain significant even when PSU fixed effects are included. Girls seem to have a lower probability of being treated with ORS by 1.3 percentage points, but the coefficient in our preferred model is only weakly statistically significant. The individual country estimates presented in **Figure 3** (panels E and F) do not follow a robust pattern. Some coefficients for the youngest and oldest age groups are negative, others are positive, and most are statistically insignificant. One exception is found for Gabon, where mothers below 20 years seem to be more likely to obtain ORS treatment for a sick child than the reference category. For Niger and Gambia we find the opposite, as mothers below 20 years seem to be less likely to obtain ORS treatment for a child with diarrhoea.

As our analysis spanned three decades, we next analysed whether the association between parents’ age and care seeking behaviour changed over time. We therefore repeated the estimations for three time periods separately. Results are summarised in **Tables 4** to **6**. The tables present coefficients for the youngest and oldest age categories only. For *care seeking for diarrhoea* and *treatment with ORS* we find a lower probability of care for children with mothers who were less than 20 years at the child’s birth during the earliest time period between 1995 and 2001. Coefficients suggest a lower probability by 11.5 and 6.2 percentage points, respectively. In this period, coefficients of father’s age suggest a higher probability of care seeking and treatment with ORS for diarrhoea among the youngest age group, by 9.1 and 7.6 percentage points, respectively.

For the more recent periods 2002-2010 and 2010-2017, these associations disappear in the preferred model specification. *Care seeking for diarrhoea* instead seems to be higher for mothers in the youngest age group and lower for fathers in the youngest age group, although these coefficients are only weakly significant. For *care seeking for ARI,* we find no significant association with mother’s or father’s age in the earliest time period. Between 2002 and 2010, a significant and negative coefficient appears for fathers above 45 years, suggesting a lower probability of care seeking by 7.6 percentage points. Between 2010 and 2017, we obtain a significant and negative coefficient for mothers below 20 years, suggesting a lower probability by 5.9 percentage points. Figures S1-S6 in the **Online Supplementary Document** display the coefficients of mother’s and father’s age from individual country analyses separately for the early time period 1995-2001 (panel a) and the most recent time period 2010-2017 (panel b). Again, we present coefficients for the youngest and oldest age group only. We do not recognise a robust pattern in the association between parents’ age and care seeking behaviour and any change in this association over time.

## DISCUSSION

In this study, we assessed the association of parents’ age with health care seeking behaviour for children who are sick with diarrhoea or ARI. The results suggest that the probability that parents seek health care when their child has fallen sick is not associated with their age. While simple models indicate an inverse U-shape pattern, there is no robust association when socio-economic characteristics and especially PSU fixed effects are included. This indicates that parents’ age might actually be a confounder of the association between socio-economic background and care seeking. In our data, children living in a household from the poorest quartile are more likely both to have a mother younger than 20 years as well as a mother older than 39 years compared to children in the richest quartile. This corresponds to similar findings for the region showing that adolescent maternity is strongly associated with lower wealth [16]. Treatment with ORS seems to be somewhat more closely associated with parents’, especially mother’s age. However, this association is not robust to including PSU fixed effects. The specific location of a household seems to absorb other factors. PSU fixed effects might partly capture access to care which is not otherwise included in the models. The mother’s level of education and the household’s wealth status seem to be more important factors for health care seeking behaviour than parents’ age. This is in line with the literature as previous studies found that both education and wealth matter for health care seeking behaviour [11,12].

Our results indicate a considerable variation in the association between parents’ age and health care seeking behaviour for the child between countries and across time. This variation between countries matches the different results for education and wealth of country-level studies as described above. The heterogeneity may also in part be driven by the different timings of surveys in each country. This is suggested by the finding that associations found for the time period 1995-2001 are not seen for later periods. While age at birth seemed to have played a role for care seeking behaviour 25 years ago, it does not seem to be as relevant today.

Our study contributes to the evidence with an analysis of the association between parental characteristics, specifically parents’ age, as well as household wealth status and health care seeking behaviour that is comparable across the included countries. Our analysis adds a further dimension by investigating whether the association changed over time.

Our study has several limitations. Above all, our analysis is limited by the available data. Previous studies indicate that survey questions used in DHS and MICS do not capture health care seeking very well [18]. A study in Nigeria found that recall of treatment with antibiotics for childhood diseases was poor in DHS and MICS (phase 5) [19]. In Pakistan and Bangladesh, only about two-thirds of caregivers recalled antibiotic treatment for childhood pneumonia correctly using DHS questions [20]. While estimating treatment rates is especially difficult due to incorrect identification of pneumonia, recall of treatment with ORS or visits to a health facility for childhood diseases may also be limited by identification of true diarrhoeal episodes. It is not clear whether our analysis would over- or underestimate actual health care seeking behaviour. In addition, the outcome variables used do not capture whether a child was treated appropriately. While giving ORS is adequate treatment of diarrhoea, seeking care outside home might not necessarily result in adequate treatment. It is rather an indication of the willingness of parents to care for a sick child. Sreeramareddy et al. use DHS and MICS to study time trends in diarrhoea case management indicators and care seeking behaviour for children and find that less than 50 percent of sick children were given correct treatment [5]. Similarly, Carvajal-Vélez et al. analyse diarrhoea management practices and find low prevalence of good diarrhoea management even if the child was taken to a health facility [21].

Another limitation of our study is that several factors that may influence care seeking are not captured in our models. These include the price of health care, access to health care, and the quality of health care facilities. We address the lack of these variables by including PSU fixed effects in our last specification as these factors should not vary within one PSU.

## CONCLUSION

Whether parents in Western and Central Africa seek care outside home for their child suffering from diarrhoea or ARI or treat diarrhoea with ORS does not seem to depend greatly on their age at birth of the child. Nevertheless, the overall likelihood that care is sought for a sick child still is rather low. Especially the likelihood of giving ORS to treat diarrhoea seems surprisingly low, given that it is a cheap and highly effective treatment.

Our study opens up questions for further research. Particularly, reasons for the low prevalence of health care seeking for childhood diseases should be investigated. We suggest adding a question in the DHS and MICS on why health care was not sought for a sick child. This would allow for an analysis of this research question using large data sets, as in this study. Answering the question on why health care seeking is low would help formulate policy and strategic response. Although the association disappeared for the more recent period, we found a positive association between mother’s age and care seeking and a negative association between father’s age and care seeking for the early time period. Investigating reasons for this difference could shed light on the different roles a mother and a father play in decisions about health care seeking for their children.

The considerable variation between countries calls for research to take into account specific country contexts. Countries implementing community Integrated Management of Childhood Illness (IMCI), for example, might follow a different trend compared to countries who do not. The variation also highlights challenges in copying strategies of addressing care seeking behaviour from best-performing countries. Strategies need to be adjusted to each context in order to be successful.

## Additional material


Online Supplementary Document
