# Supplementary material for: Health-care seeking for childhood diseases by parental age in Western and Central Africa between 1995 and 2017: A descriptive analysis using DHS and MICS from 23 low- and middle-income countries
Source: J Glob Health. 2021 Aug 10;11:13010. doi: 10.7189/jogh.11.13010 (PMC8397328; doi:10.7189/jogh.11.13010)
Supplement: Online Supplementary Document [file jogh-11-13010-s001.pdf]

Figures S1 – S6. Coefficients of parents' age from model 3 (with father's age) by country, separate for the first period 1995-2001 and the most recent period 2010-2017

Figure S1. Coefficients of mother's age by country for first and most recent time period, outcome variable: care seeking for diarrhoea

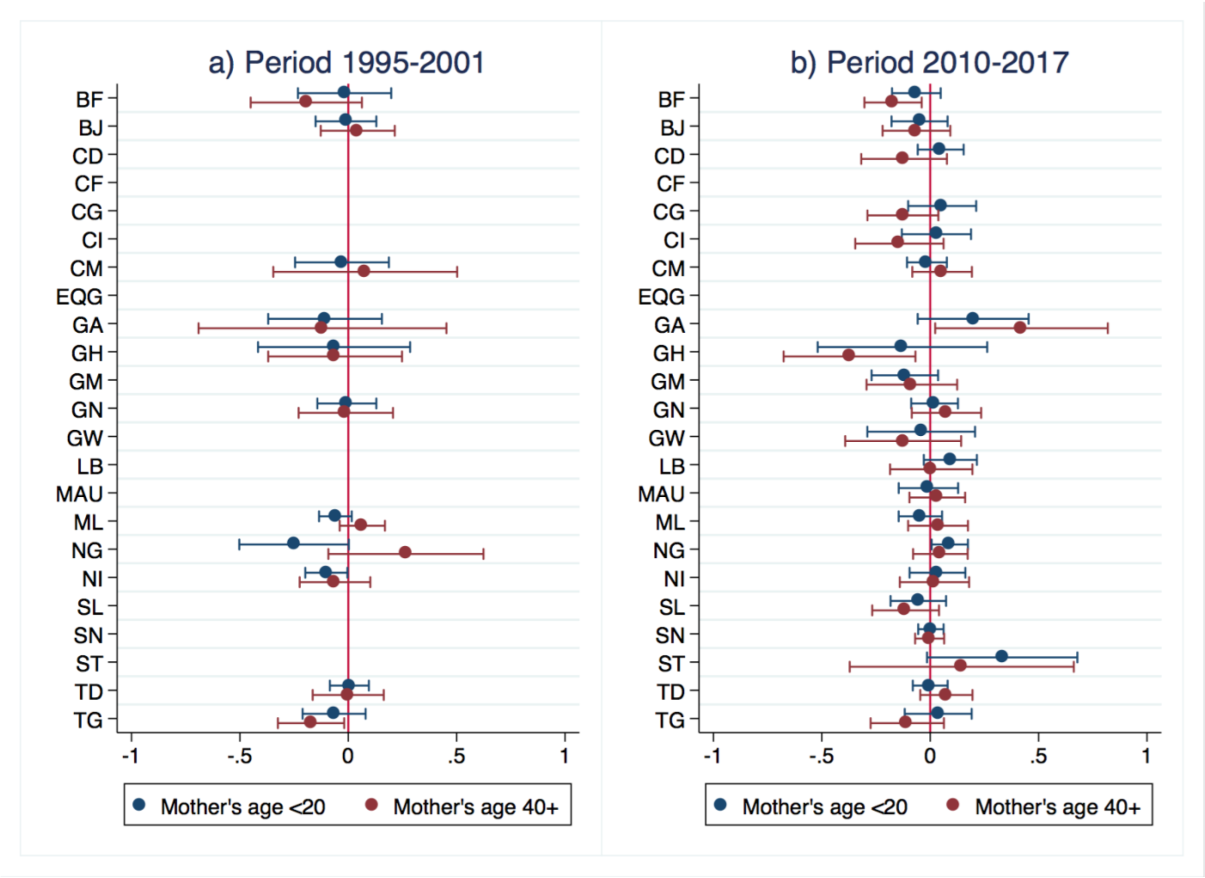

Note: Coefficients and confidence intervals of mother's age, age categories <20 and 40+, based on country-specific regressions following column (7) in **Table 4**, panel 1 and 3.

Figure S2. Coefficients of father's age by country for first and most recent time period, outcome variable: care seeking for diarrhoea

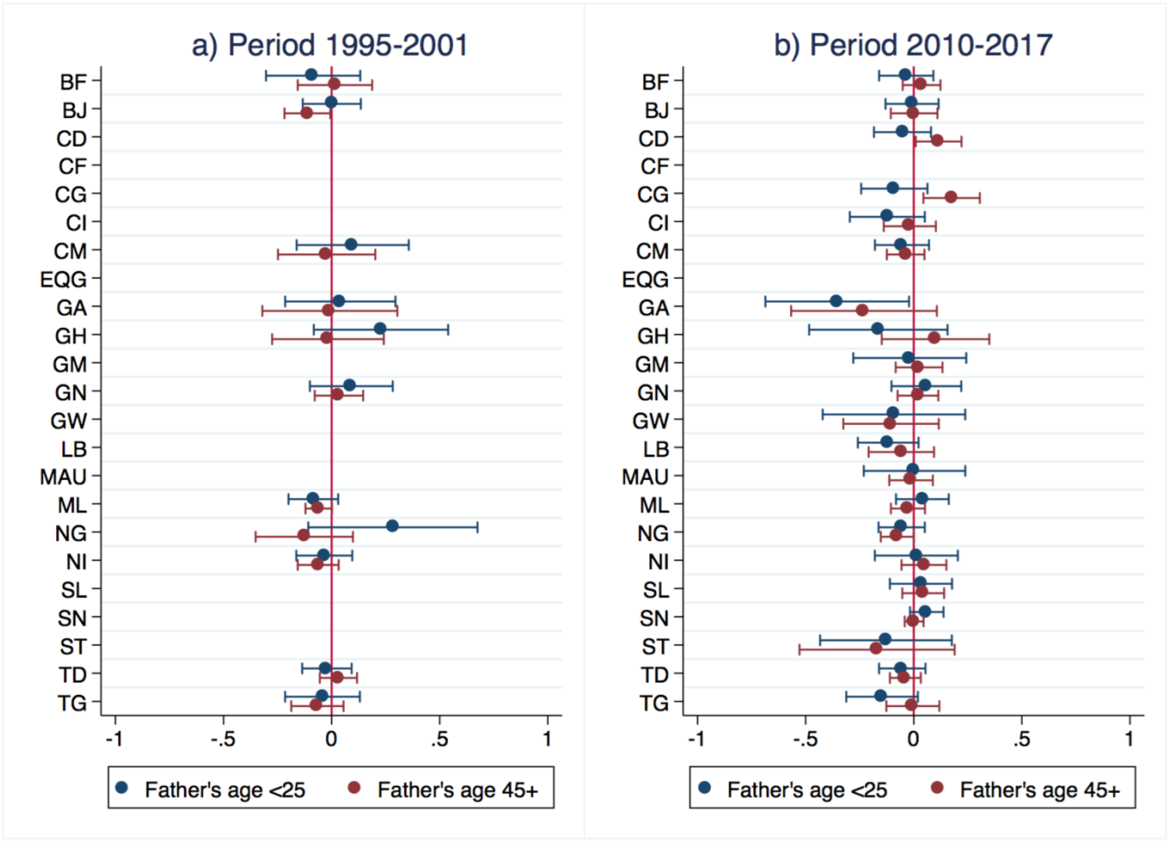

Note: Coefficients and confidence intervals of father's age, age categories <25 and 45+, based on country-specific regressions following column (7) in **Table 4**, panel 1 and 3.

Figure S3. Coefficients of mother's age by country for first and most recent time period, outcome variable: care seeking for ARI

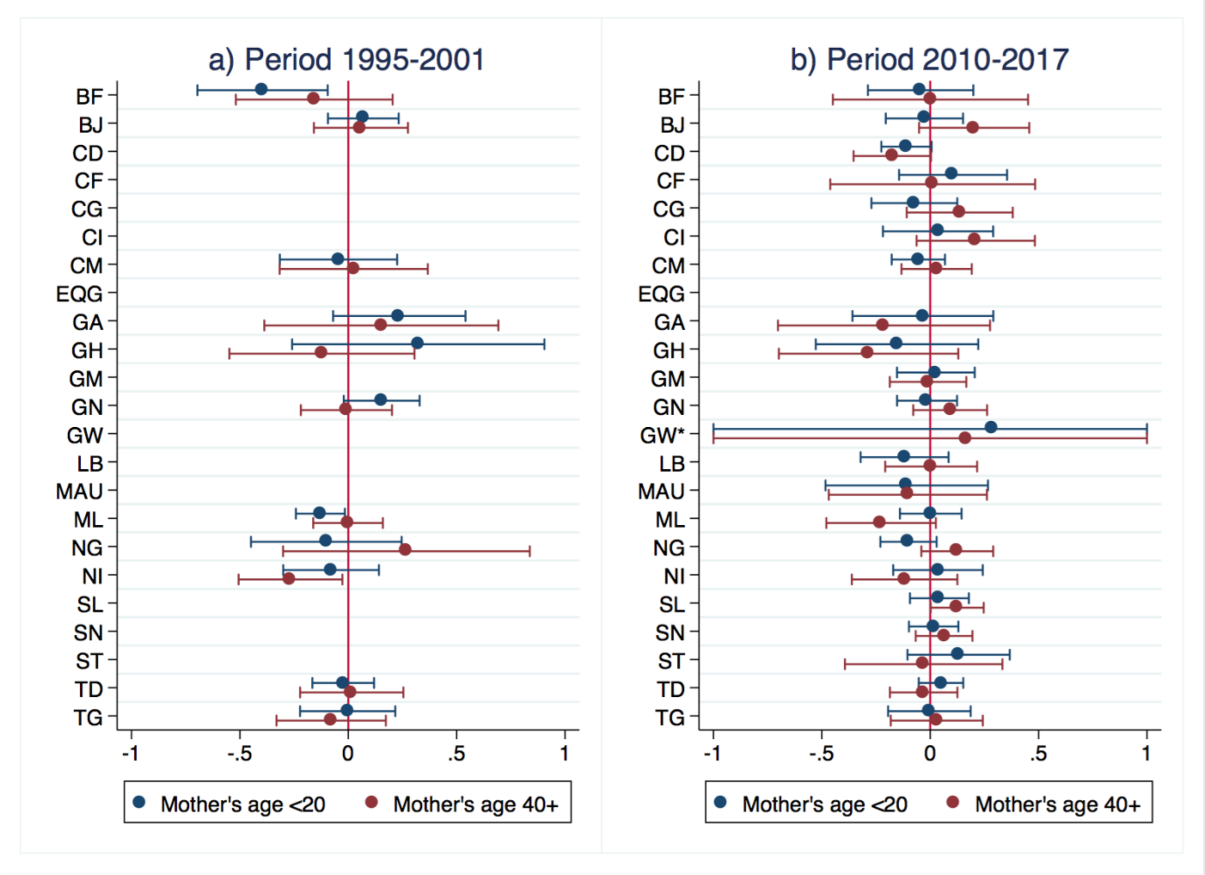

Note: Coefficients and confidence intervals of mother's age, age categories <20 and 40+, based on country-specific regressions following column (7) in **Table 5**, panel 1 and 3. *The confidence intervals for Guinea-Bissau (2010-2017) exceed -1 and 1, but were shorted to fit into the graph.

Figure S4. Coefficients of father's age by country for first and most recent time period, outcome variable: care seeking for ARI

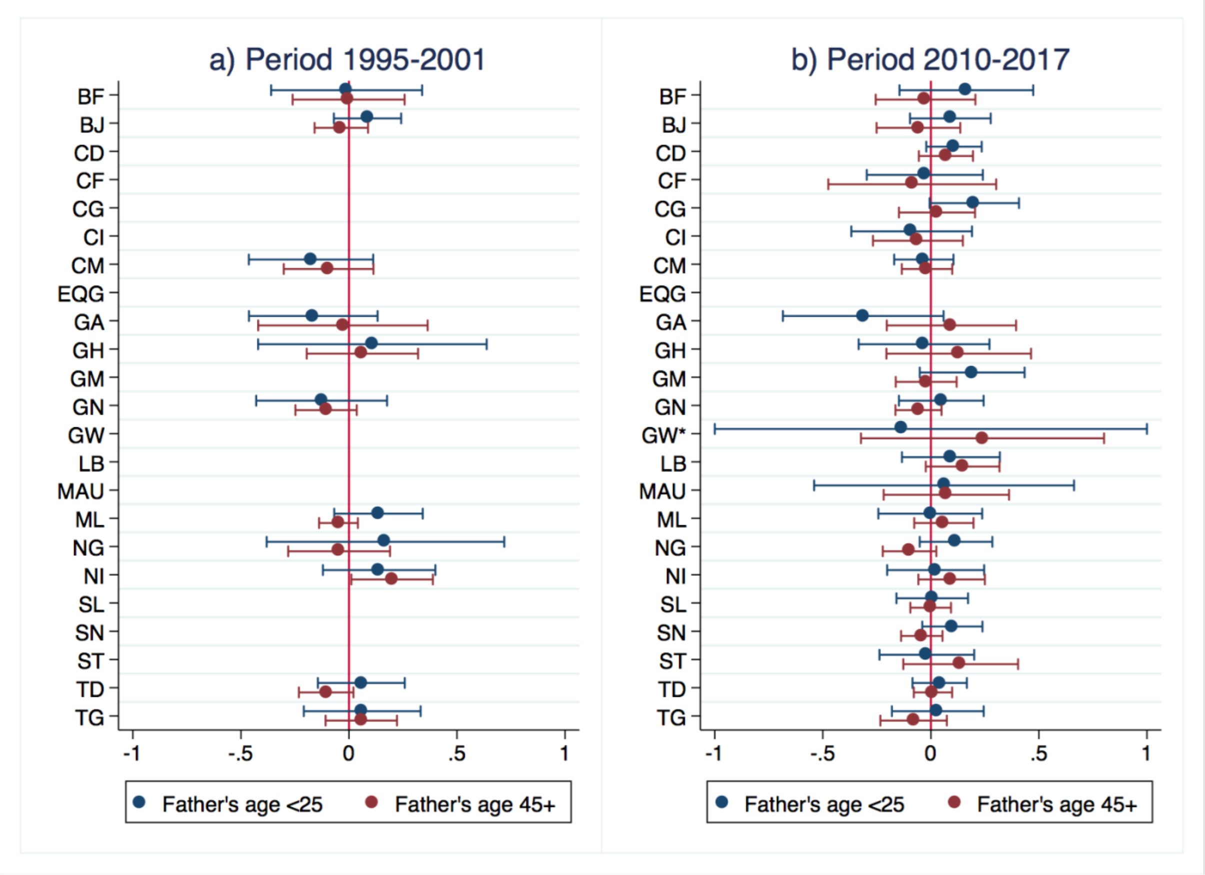

Note: Coefficients and confidence intervals of father's age, age categories <25 and 45+, based on country-specific regressions following column (7) in **Table 5**, panel 1 and 3. *The confidence intervals for Guinea-Bissau (2010-2017), age group <25, exceed -1 and 1, but were shorted to fit into the graph.

Figures S5. Coefficients of mother's age by country for first and most recent time period, outcome variable: treatment with ORS

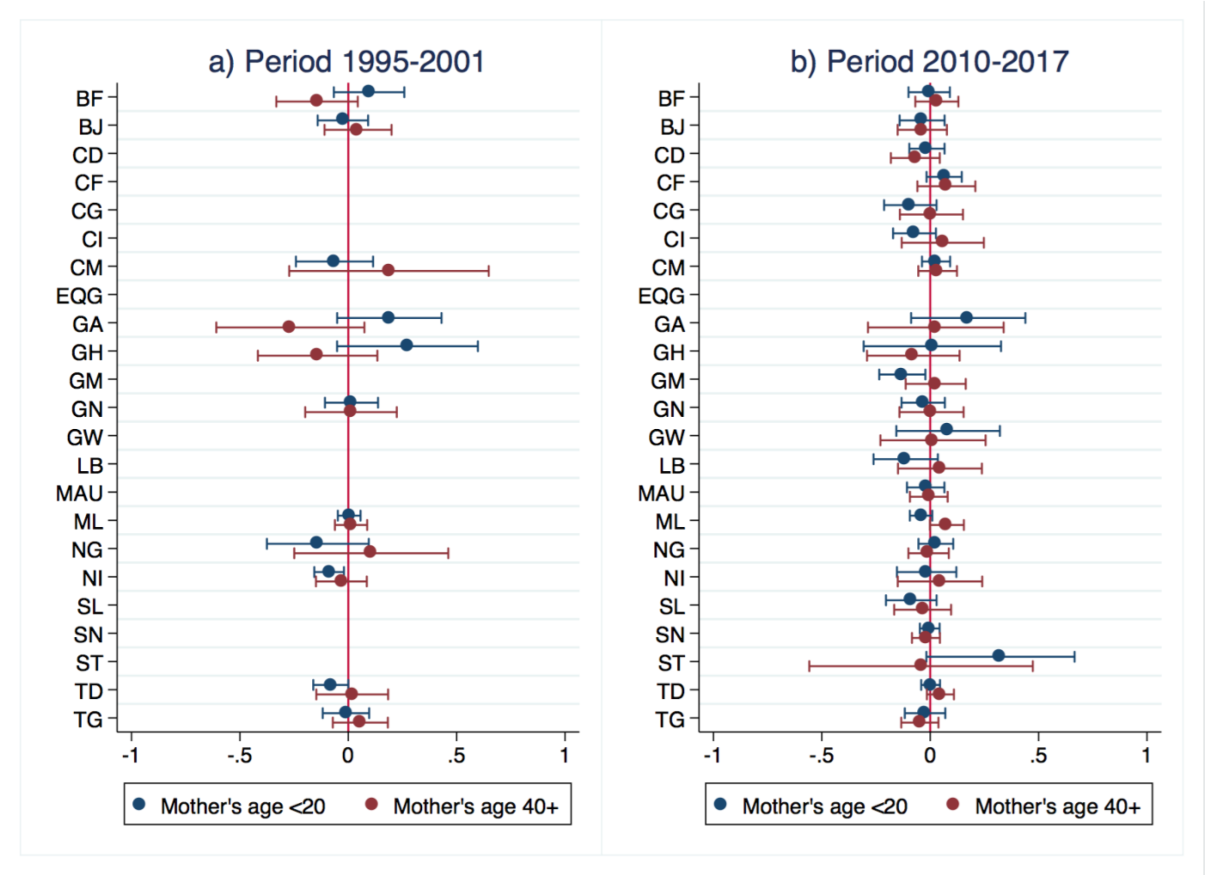

Note: Coefficients and confidence intervals of mother's age, age categories <20 and 40+, based on country-specific regressions following column (7) in **Table 6**, panel 1 and 3.

Figure S6. Coefficients of father's age by country for first and most recent time period, outcome variable: treatment with ORS

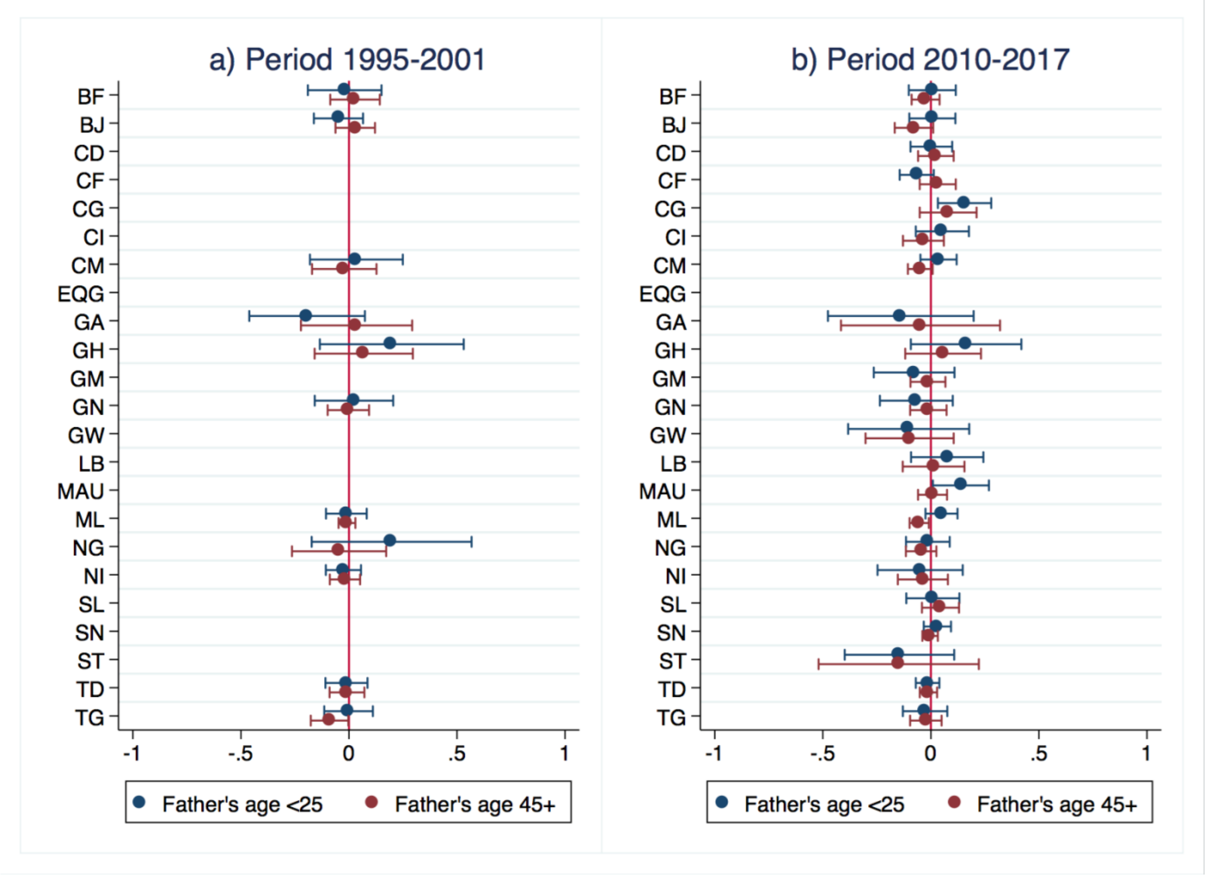

Note: Coefficients and confidence intervals of father's age, age categories <25 and 45+, based on country-specific regressions following column (7) in **Table 6**, panel 1 and 3.
